# Reticulated Platelets in Medicine: Current Evidence and Further Perspectives

**DOI:** 10.3390/jcm9113737

**Published:** 2020-11-20

**Authors:** Noé Corpataux, Kilian Franke, Alexander Kille, Christian Marc Valina, Franz-Josef Neumann, Thomas Nührenberg, Willibald Hochholzer

**Affiliations:** Klinik für Kardiologie und Angiologie II, Universitäres Herzzentrum Freiburg, 79189 Bad Krozingen, Germany; noe.corpataux@universitaets-herzzentrum.de (N.C.); alexander.kille@universitaets-herzzentrum.de (A.K.); christian.valina@universitaets-herzzentrum.de (C.M.V.); franz-josef.neumann@universitaets-herzzentrum.de (F.-J.N.); thomas.nuehrenberg@universitaets-herzzentrum.de (T.N.); willibald.hochholzer@universitaets-herzzentrum.de (W.H.)

**Keywords:** immature platelets, reticulated platelets, cardiology, percutaneous interventions, bleeding, thrombocytopenia

## Abstract

Reticulated platelets (RPs) are young thrombocytes, newly released from the bone marrow. The identification and quantification of these cells remained difficult for decades due to a lack of standardized preanalytical and analytical methods. With the introduction of automated hematology analyzers in clinical routine, the determination of RPs, either as a total count or as a fraction, became more reliable, faster and more affordable. Currently, RPs are the focus of research in multiple clinical settings. In cardiovascular medicine, recent studies have focused on the relationship between RPs, coronary artery disease (CAD) and clinical outcomes, as well as the impact of RPs on the effects of antiplatelet therapy. Cohort studies showed increased levels of RPs in patients with acute coronary syndrome (ACS) or cardioembolic stroke. In patients with ACS, increased levels of RPs were also associated with an increased incidence of major ischemic cardiovascular events during follow-up. Further studies showed an association of levels of RPs with the antiplatelet response to less-potent P2Y12 inhibitors. In patients with paroxysmal atrial fibrillation undergoing pulmonary vein isolation, levels of RPs differed significantly depending on the achieved rhythm (sinus rhythm vs. recurrent atrial fibrillation). Levels of RPs appear to also be predictive for bleeding events in patients with various hematological diagnoses. Although no causal relationship has so far been proven, RP values have been associated with a large number of pathologies and clinical scenarios. This review summarizes the current evidence with regard to RPs and their potential diagnostic and prognostic value for noncardiovascular patients and for cardiovascular patients in particular. It describes further perspectives on how the testing of these cells might improve the treatment of cardiovascular patients.

## 1. Introduction

**Figure 1:**
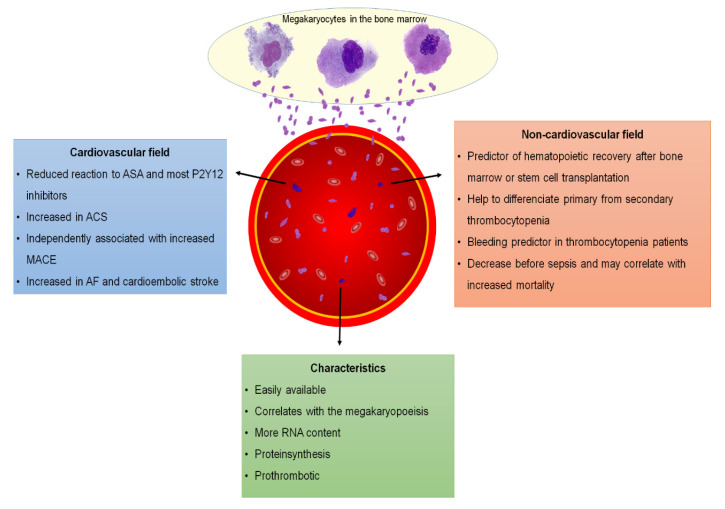
Characteristics of immature platelets and their clinical significance. ACS = acute coronary syndrome; AF = atrial fibrillation; ASA = acetylsalicylic acid; RNA = ribonucleic acid.

Reticulated platelets (RPs) are platelets which have been newly released from the bone marrow. They were first described in 1969 by Ingram and Coopersmith, who noticed a previously unreported type of platelet with punctate condensations, which was found in dogs following acute blood loss. In analogy to reticulocytes in erythropoiesis, these platelets were named reticulated platelets [1,2]. These cells, which according to various studies might have an increased reactivity, are larger than mature platelets and contain more ribonucleic acid (RNA) (Figure 1), which was thought to be a vestigial remnant of megakaryocytic RNA [3,4].

Recent studies have demonstrated that these platelets also contain the rough endoplasmic reticulum needed for protein synthesis and that they can regulate the biosynthesis of a selected number of proteins, indicating that their mRNA has a functional relevance [5]. RPs persist for 24 to 36 h in the circulatory system; during this period, their RNA progressively degrades and the cell size decreases, until they became “regular” platelets [6,7]. Given these results, RPs are thought to reflect the activity of megakaryopoiesis in bone marrow and may serve as a marker of platelet turnover [8]. Over the last few years, the interest in RPs has increased, partially because this subfraction of platelets is thought to have a greater functional potential than mature platelets due to their capacity for protein synthesis [9]. This review summarizes the current evidence with regard to RPs, their potential diagnostic and prognostic value for noncardiovascular patients, and for cardiovascular patients in particular. It describes further perspectives on how the testing of these cells might improve the treatment of cardiovascular patients.

## 2. Detection and Quantification of Reticulated Platelets

**Figure 2:**
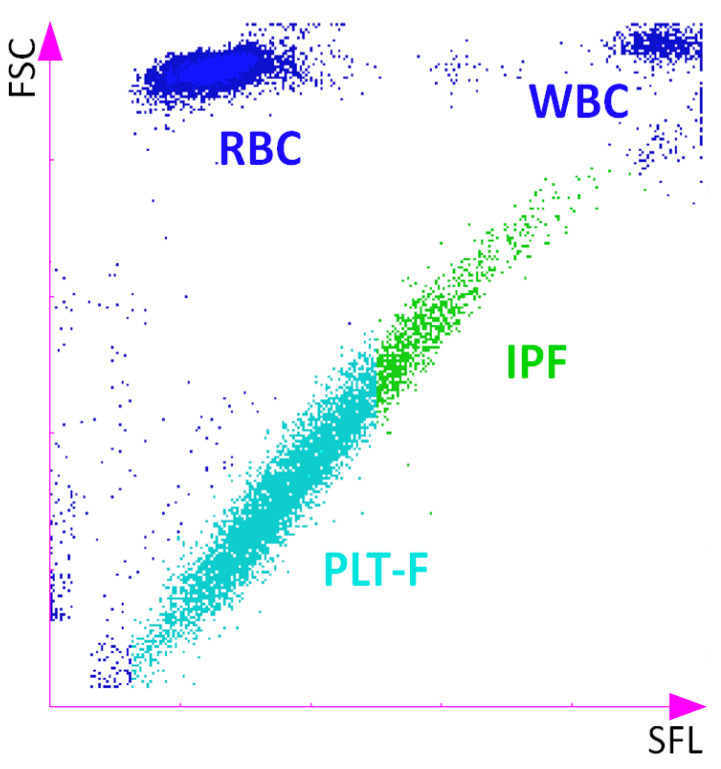
Scatterplot of a Sysmex XN-series cell counter, measuring the immature platelet fraction (IPF) in the platelet fluorescence (PLT-F) channel using forward-scattered light (cell volume) against side fluorescence (nucleic acid amount). FSC = forward-scattered light; RBC = red blood cell; SFL = side fluorescence; WBC = white blood cell.

At the time of their first description in 1969, Ingram and Coopersmith used a methylene blue dye to differentiate cells and discovered RPs as a subfraction of the platelet pool [1]. At about the same time, Karpatkin had already described differently sized platelet populations and assumed that they might have different functions [2]. Later on, the more RNA-specific dye thiazole orange became standard for the detection of these cells since it offered additional options for analysis with respect to size, count and function. Together with the use of flow cytometry, it was possible to prove the association of levels of RPs with the reproductive function of the bone marrow [8,10,11]. The lack of standardized test protocols made it hard to compare study results and limited the clinical utility of these cells as potential diagnostic and prognostic markers [12,13]. In recent years, several medical product companies (e.g., Sysmex Corporation, Kobe, Japan; Abbott, Abbott Park, IL, USA; Mindray, Shenzen, P. R. China) have introduced fully automated hematology analyzers to count RPs by using RNA-specific fluorescent dyes, which allow more standardized measurements. A sample of such a standardized algorithm for RP detection is shown in Figure 2. 

There are several different parameters used by fully automated hematology analyzers to describe the RP population, such as the immature platelet count (IPC, the absolute count of immature platelets) and the immature platelet fraction (IPF, the percentage of immature platelets in relation to the overall platelet count) [14]. In the literature, these acronyms, as well the common term “immature platelets” (IPs), are used to describe RPs. The current key limitation is that the results of automated hematology analyzers from different companies do not completely match, and there is no consensus regarding the optimal parameter for RPs (e.g., absolute count or fraction). These two measurable RP indices give contrasting results in various studies, and do not seem to react in unison [15,16,17]. A recent study comparing different RP parameters for the prediction of pharmacodynamic responses to antiplatelet drugs showed that the representation of RPs using the absolute count might be the optimal parameter, as compared to parameters representing the fraction of the overall count [14]. Even if these results between the different automated analyzers are not interchangeable, the key advantage of hematology analyzers is the ubiquitous and affordable availability of RP results, thus allowing the use of this biomarker in clinical routine [18]. Since the testing of RPs with fully automatized analyzers does not allow the downstream analysis of RPs, alternative methods for the sorting of RNA-rich platelets are needed, particularly for research purposes [10,18,19]. One RNA dye which has shown potential for this purpose is SYTO 13, since it offers several advantages when compared to thiazole orange [10,19]. SYTO 13 staining remains stable over time, allowing longer sorting times, and has shown a good correlation with the fraction values from the Sysmex automated analyzer, which is not the case for thiazole orange [20]. While these more sophisticated methods enable the analysis of specific features of RPs such as a prothrombotic transcriptomic profile, the automated determination of RPs appears to be the preferable quantification method for clinical use given its highly standardized testing approach, which allows the comparison of results between different cohorts [19,21].

## 3. Immature Platelets and Noncardiovascular Diseases

RPs were first used in clinical decision-making for hematologic disorders, where these cells were used as a surrogate parameter of megakaryopoiesis in order to differentiate primary from secondary thrombocytopenia. RPs, represented as a fraction, increase in diseases with peripheral platelet destruction, such as immune thrombocytopenia or acute blood loss. In primary thrombocytopenia with decreased platelet production in the bone marrow as a central mechanism, normal or decreased levels of RPs have been found [20,22,23,24]. Levels of RPs were also found to be better predictors of significant bleeding than the platelet count in a cohort of 97 pediatric patients with immune thrombocytopenia. Higher levels of RPs were associated with a decreased risk of severe or life-threatening hemorrhage in patients with a very low platelet count [25]. These findings are in line with the results of a prospective study analyzing the risk of bleeding in a cohort of adult patients with a wide range of different hematological disorders. In this cohort, the RPs were correlated with the risk of bleeding (WHO grade ≥ 2) within 1 day of blood sampling [26]. More recently, RPs together with schistocyte counts were found to be discriminative between two overlapping diagnoses causing thrombotic microangiopathy in pregnancy: severe preeclampsia/hemolysis, elevated liver enzymes and low platelet syndrome (SPE/HELLP) and thrombotic thrombocytopenic purpura [27].

Regarding platelet production recovery, RPs were found to be a reliable prognostic tool after bone marrow or stem cell transplantation. In these patients, an increase in RPs was seen several days before an increase in the total platelet count occurred. This has been shown to be one of the earliest predictors of hematopoietic recovery following bone marrow and stem cell transplantation [28,29,30]. A very interesting observation coming from a trial with 40 pediatric patients with hematological disorders demonstrated that prophylactic platelet transfusions with concentrates containing high percentages of RPs seem to be more effective than concentrates with low percentages of RPs, leading to a lower number of required transfusions [31]. Implementing this laboratory parameter in transfusion strategies and in the thrombocytopenic phase following chemotherapy has the potential to reduce the required number of platelet transfusions, which is currently mainly based on the platelet count alone. 

Severe infections and sepsis are another field where RPs might serve as a diagnostic and prognostic marker. De Blasi and colleagues showed an increase in RPs 2 to 3 days before the appearance of symptoms in patients with sepsis, followed by a decrease after the appearance of symptoms, indicating a potential use of this parameter as an early diagnostic marker [32]. A number of studies have demonstrated the association of increased RP levels with an increased 28-day mortality and sepsis severity, generally assumed to be associated with elevated thrombocytes destruction within an ongoing coagulopathy [33,34]. On the other hand, Koyama et al. showed that a significant decrease in RPs in patients with sepsis was associated with increased mortality within 28 days, possibly reflecting the impaired bone marrow function in severe sepsis [35]. The systematic review of Thorup and colleagues, including 14 observational studies with a total of 2509 septic patients, showed mixed results. While most studies have suggested that an increased level of RPs is directly associated with disease severity and mortality in patients with sepsis and septic shock, some did not find these associations [36]. This could be explained by different disease severity at baseline or by the different designs of the studies. Recent research on patients hospitalized with SARS-CoV-2 infection also showed elevated RP levels in these patients compared to stable patients with cardiovascular risk factors. During progressive SARS-CoV-2 infection, levels of RPs were even higher as compared to patients with acute myocardial infarction. These findings might indicate a potential pathway between RPs and thrombotic events in COVID-19 patients [17]. Table 1 provides an overview of the most relevant noncardiovascular studies.

## 4. Immature Platelets and Cardiovascular Disease

In 2004, Lakkis et al. were the first group to show elevated levels of RPs in patients with acute coronary syndrome (ACS) as compared to patients with stable angina [39]. Following studies focused on the potential interaction of RPs and response to antiplatelet drugs, since an impaired antiplatelet response has been associated with adverse cardiovascular outcomes, particularly following percutaneous coronary intervention [14,40,41,42]. A small study enrolling 60 healthy volunteers demonstrated that higher levels of RPs were associated with increased platelet aggregation and activity and diminished antiplatelet effects of aspirin [4]. These results were also seen in a larger population of patients with stable coronary artery disease (CAD). Higher levels of RPs have been associated with increased platelet aggregation in dual antiplatelet therapy with aspirin and clopidogrel [43]. A similar association of impaired antiplatelet response and RP levels was demonstrated in a small cohort of patients presenting with ST-elevation myocardial infarction (STEMI) for prasugrel [44] but not for ticagrelor [3]. As a potential explanation for this finding for ticagrelor, its different metabolism and receptor was discussed in comparison to thienopyridines such as clopidogrel or prasugrel, which bind the P2Y12 receptor of platelets irreversibly. In contrast, ticagrelor is an active drug that binds reversibly with the P2Y12 receptor and results in a continuous circulation of active reversibly binding ticagrelor in the blood flow, which might allow continuous inhibition of RPs freshly released from the bone marrow [3]. For prasugrel and clopidogrel, it was suggested that the association of levels of RPs and impaired antiplatelet response was caused by decreased drug exposure due to high platelet turnover [45]. However, this hypothesis could not be confirmed in a later study with a larger cohort, which demonstrated a similar association of immature platelet count with antiplatelet response to thienopyridines, early and late after intake of the drug. Thus, the main underlying mechanism could be another effect of RPs on thienopyridines, such as one resulting from the intrinsic properties of RPs [46]. Since the association of levels of RPs with an antiplatelet response to P2Y12 receptor inhibitors was strongest for the less-potent clopidogrel, the antiplatelet potency of the inhibitor might also be an important factor for the strength of this association. A further study investigating the impact of levels of RPs on the antiplatelet effect of the strong intravenous P2Y12 receptor inhibitor cangrelor might support this hypothesis, since no correlation between RPs and platelet reactivity could be found for cangrelor [47]. 

Several studies have investigated the potential value of RPs for diagnosis and prognosis in patients with CAD. The Novara Atherosclerosis Study enrolled 1789 patients. The key finding was that the IPF was not associated with the prevalence and extent of CAD among patients undergoing coronary angiography [48]. Several subanalyses of this study showed no association of levels of RPs with concomitant antiplatelet therapy, use of statins, age or gender [49,50]. Only in the subgroup of patients with CAD and diabetes mellitus were high levels of RPs associated with a lower prevalence of CAD [51], but with a higher incidence of major adverse cardiovascular events and a lower incidence of bleeding [52]. Several studies comparing healthy subjects, patients with stable CAD and patients with ACS showed significant elevated levels of RPs in patients with ACS, especially in patients with STEMI [39,53,54]. However, a later study demonstrated that the IPF and mean platelet volume did not improve the diagnosis of patients with suspected ACS presenting to the emergency department [55]. With respect to the prognostic value of RPs, a subanalysis of the prospective AMI-Florence 2 Study, which included consecutive patients with suspected STEMI, demonstrated for the first time that the IPF is an independent predictor of cardiovascular death within 12 months in patients with ACS undergoing percutaneous coronary intervention (PCI) [56]. Another study enrolling 89 patients with CAD and following patients for a median time of 31 months demonstrated that the IPC is a predictor for the occurrence of the composite endpoint of all-cause mortality, myocardial infarction, unplanned revascularization or recurrent angina requiring hospitalization [57]. These findings were confirmed in a prospective study with 477 patients undergoing PCI and a median follow-up period of 5.8 years, in which increased platelet turnover was associated with a long-term adverse outcome [58]. 

In patients undergoing major noncardiac surgery, preoperative increased levels of RPs were associated with a higher 30-day mortality and a higher rate of postoperative myocardial injury [59].

For nonvalvular atrial fibrillation, which is associated with an increased risk of thromboembolic events [60], two studies described a significant decrease in levels of RPs for 3 to 4 months following pulmonary vein isolation in patients with stable sinus rhythm in comparison to patients with atrial fibrillation [61,62]. In patients with cardioembolic stroke, significantly higher levels of RPs were found as compared to control patients [63]. For patients with ischemic cerebrovascular disease, an age-dependent increase in levels of RPs was found in the early and late phases of stroke [64]. Taken together, these data suggest that RPs might play a major role in the increased thrombotic activity seen in ischemic disorders regardless of the antiplatelet therapy used. 

A summary of the major studies in the cardiovascular field is presented in Table 2.

## 5. Future Perspectives

The currently available data indicate that immature platelets may serve as an important diagnostic and prognostic parameter not only in cardiovascular medicine but also in other settings, such as hematology or severe infections. Given the recent advances regarding standardization in the testing of RPs, they can now be seen as a ubiquitously available and affordable laboratory parameter, thus enabling their widespread use in clinical routine. However, before the testing of RPs can enter clinical routine, further research is needed to determine the optimal parameter (total count or proportion) and optimal cutoffs for RPs, and to evaluate the algorithms required for improved clinical decision-making. 

The exact mechanisms of altered levels of RPs and the variation of these levels remain unknown. While strong associations have been demonstrated in the numerous studies cited in this review, no direct causality has been demonstrated despite various hypotheses. Other unsolved questions with respect to RPs include the temporal course of this parameter, in particular following conditions such as ACS, the prognostic impact of these changes and potential interaction of medication or medical treatments on levels of RPs. In conclusion, given the currently available data, testing of RPs might improve the early diagnosis of acute diseases such as ACS and severe infections, and may improve the risk assessment of patients. Since the currently available tools for the prediction of bleeding and ischemic events have multiple limitations, including limited sensitivity, the testing of RPs might be a potential approach to a more personalized precision medicine.

## Figures and Tables

**Table 1:** Overview of the different studies of importance outside the cardiovascular field.

Authors	Year of Publication	No. of Patients	Study Setting Summary
**Reticulated platelets in hematological disorders**			
C. Briggs et al. [22]	2004	Control group: 50	Assessment of RPs in peripheral thrombocytopenia
		Study group: 22	
M.L. Zucker et al. [28]	2006	50	RPs used to predict platelet recovery after hematopoietic progenitor cell transplantation
A. Takami et al. [29]	2007	25	RPs for the prediction of platelet engraftment after allogenic stem cell transplantation
N. Sugimori et al. [37]	2009	Control group: 170	RPs in patients with myelodysplastic syndrome
		Study group: 71	
H. Jung et al. [24]	2010	Control group: 1837	Determining the reference intervals of RPs and the optimal cutoff value to differentiate ITP from AA
		Study group: 202	
G. Strauss et al. [20]	2010	87	RPs in distinguishing ITP from ALL in pediatric patients
N. van der Linden et al. [30]	2014	18	RPs used to predict platelet recovery after autologous stem cell transplantation
M. Sakuragi et al. [23]	2015	Control group: 80	RPs for distinguishing ITP from aplastic thrombocytopenic disorders
		Study group: 75	
A. McDonnell et al. [25]	2018	272	RPs in pediatric patients to differentiate ITP from bone marrow failure and predict bleeding score
R.A. El-Gamal Fayek et al. [27]	2019	73	RP and schistocyte count in pregnant women with ITP and SPE/HELLP
M.J. Jeon et al. [38]	2020	568	RP predictive scoring model for ITP
**Reticulated platelets in sepsis**			
De Blasi, R.A., et al. [32]	2013	64	RPs in predicting sepsis in critically ill patients
Enz Hubert RM et al. [34]	2015	41	Association of RPs with sepsis diagnosis and severity
T. Muronoi et al. [33]	2016	149	RPs in predicting mortality in patients with sepsis
Koyama K. et al. [35]	2018	205	RPs and their relation to thrombocytopenia and mortality in patients with sepsis
**Reticulated platelets in SARS-CoV-2 infections**			
Cohen A. [17]	2020	Control group: 164 Study group: 47	RPs in patients hospitalized with COVID-19

The automated method (Sysmex) was used for RP counts in all the reported studies. AA = aplastic anemia; ALL = acute lymphocytic leukemia; ITP = idiopathic thrombocytopenic purpura; RP = reticulated platelet.

**Table 2:** Overview of the different studies of importance in the cardiovascular field.

Authors	Year of Publication	No. of Patients	RP Count Method	Study Setting Summary
**Reticulated platelets and coronary artery disease**				
N. Lakkis et al. [39]	2004	Control group: 13	Nonautomated	RPs in ACS
		Study group: 79		
F. Cesari et al. [56]	2013	229	Automated (Sysmex)	RPs predicting cardiovascular death in ACS
L. Perl et al. [52]	2019	104	Automated (NA)	Prognostic significance of RP levels in DM patients with stable CAD
NAS [48,49,50,51]	2017	1789	Automated (Sysmex)	Assessing the relationship between RPs and the prevalence and extent of CAD
	2020	2236	Automated (Sysmex)	Impact of aging on RP count and its relationship with CAD
	2020	1781	Automated (Sysmex)	Impact of DM on RPs and its association with CAD
	2020	2550	Automated (Sysmex)	Impact of gender on RP count and its relationship with CAD
**Reticulated platelets and antiplatelet therapy**				
S. Guthikonda et al. [4]	2007	60	Nonautomated	RPs and the effects of ASA
S. Guthikonda et al. [43]	2008	90	Nonautomated	Role of RPs in platelet activity after DAPT with clopidogrel and ASA
F. Cesari et al. [45,56]	2008	372	Automated (Sysmex)	Role of RPs in platelet function of high-risk patients under DAPT
L. Perl et al. [44,52]	2014	62	Nonautomated	Association between RPs and platelet aggregation under prasugrel
I. Bernlochner et al. [3]	2015	124	Automated (Sysmex)	Association between RPs and platelet aggregation under prasugrel or ticagrelor
C. Stratz et al. [46]	2016	199	Automated (Sysmex)	Impact of RPs on antiplatelet response to thienopyridines
C. Stratz et al. [47]	2018	110	Automated (Sysmex)	Impact of RPs on antiplatelet effect of cangrelor
NAS [65,66]	2018	286	Automated (Sysmex)	Impact of long-term DAPT on RP count and platelet reactivity
	2019	1475	Automated (Sysmex)	Impact of long-term ASA on RP count
**Reticulated platelets and cardiovascular outcomes**				
H. Ibrahim et al. [57]	2014	89	Automated (Sysmex)	Association of RPs with adverse cardiovascular outcomes
M. Tscharre et al. [58]	2019	477	Automated (Sysmex)	Impact of RPs on long-term adverse cardiovascular outcomes
A.J.A. Meershoek et al. [59]	2020	2971	Automated (Abbott)	RPs as predictors of MI and mortality after noncardiac surgery

ACS = acute coronary syndrome; ASA = acetylsalicylic acid; CAD = coronary artery disease; DAPT = dual antiplatelet therapy; DM = diabetes mellitus; RPs = reticulated platelets; MI = myocardial injury; NA = not available; NAS = Novara Atherosclerosis Study Group.
